# Primitive Basic Amino Acids Promote Mineral-Catalyzed Electrochemical Reduction of H^+^ and CO_2_

**DOI:** 10.1021/acs.jpcc.6c02384

**Published:** 2026-06-18

**Authors:** Siang Chen, Tatsuya Corlett, Norio Kitadai, Ryuhei Nakamura, Masahiro Miyauchi, Liam M. Longo, Akira Yamaguchi

**Affiliations:** 1 Department of Materials Science and Engineering, School of Materials and Chemical Technology, 13290 Institute of Science Tokyo, 2-12-1 Ookayama, Meguro-ku, Tokyo 152-8552, Japan; 2 Earth-Life Science Institute, 13290 Institute of Science Tokyo, Tokyo 152-8550, Japan; 3 Super-cutting-edge Grand and Advanced Research (SUGAR) Program, 13570 Japan Agency for Marine-Earth Science and Technology (JAMSTEC), 2-15 Natsushima-cho, Yokosuka 237-0061, Japan; 4 Biofunctional Catalyst Research Team, RIKEN Center for Sustainable Resource Science, 2-1 Hirosawa, Wako, Saitama 351-0198, Japan; 5 Department of Chemical Science and Engineering, School of Materials and Chemical Technology, 13290 Institute of Science Tokyo, 2-12-1 Ookayama, Meguro-ku, Tokyo 152-8552, TokyoJapan; 6 Hydrogen Boride Research Center, Tsukuba Institute of Advanced Research, University of Tsukuba, 1-1-1 Tennodai, Tsukuba, Ibaraki 305-8573, Japan; 7 Blue Marble Space Institute of Science, Seattle, Washington 98154, United States

## Abstract

Early in the history of life, mineral surfaces, such as the NiFeS mineral violarite (FeNi_2_S_4_), may have been the primary catalysts. How these primitive chemical systems could have undergone robust chemical evolution remains unknown. Here, we show that simple amino acids, such as diaminobutyric acid (DAB), can accelerate H^+^ and/or CO_2_ electrochemical reduction on violarite. Reactions performed with structurally similar compounds demonstrate that both amino groups of DAB participate in HER catalytic enhancement. Spectroscopic analyses suggest that the role of DAB is to deliver protons to the mineral surface. The observation that simple basic amino acids can enhance surface-supported electrochemistry reveals that amino acids could have produced a positive feedback that promoted metabolic complexification even before their eventual incorporation into complex peptide or protein catalysts.

## Graphical abstract

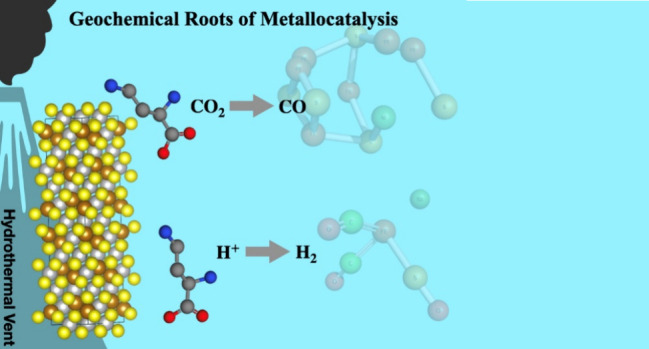

## Introduction

At the earliest stages of biological evolution, mineral surfaces subject to electrochemical gradients may have served as key catalysts,^1–4^ promoting the production of molecular hydrogen (hydrogen evolution reaction, HER) and reducing CO_2_ (carbon dioxide reduction reaction, CRR). When coupled with geochemical processes like serpentinization, these reactions could have provided both the energy and the chemical precursors necessary to sustain primitive life. In addition to supporting CO_2_ reduction into organic compounds, H_2_ generation may have also promoted the formation of minerals such as awaruite (Ni_3_Fe), which are capable of catalyzing the formation of metabolites such as methane, formate, and acetate.^5^ While serpentinization readily produces H_2_, it consumes the mineral reductant (olivine and pyroxenes) in the process, and is thus self-limiting. Electrochemical H_2_ evolution, on the other hand, has the advantage that it is a *catalytic* process that does not consume the participating mineral. Thus, as long as electrons, protons, and potential energy are available, surface-catalyzed reduction reactions can proceed. This potential difference in longevity may be an important factor for the emergence of proto-metabolism.

Contemporary metabolic networks contain autocatalytic loops.^6,7^ For example, the formation of tetrahydrofolate (THF) can require THF as a cofactor.^8^ Likewise, purine synthesis requires purine-derived cofactors.^9^ Crucially, these autocatalytic loops can impart exponential growth potential to a metabolic network, meaning that these systems can capture flux more efficiently as they grow.^10^ Here, we explore this idea from the perspective of heterogeneous catalysis: could simple metabolites, plausibly downstream of the HER and CRR reactions supporting a primitive metabolism, interact with the mineral surface in such a way as to promote HER and CRR?

We focus our attention on violarite (FeNi_2_S_4_), an iron–nickel sulfide mineral that may have served as a potential catalyst in prebiotic reactions.^11–13^ The composition of violarite resembles active sites of metalloenzymes like [Ni–Fe] hydrogenase^14^ and carbon monoxide dehydrogenase (CODH),^15^ which are central to HER and CRR catalysis in cells, respectively. Though this similarity has been taken to suggest a kind of continuity between mineral surfaces and metal clusters in enzymes, this interpretation has recently been challenged.^200^

Previously, we have shown that the amino acid l-histidine can enhance CRR activity on violarite surfaces, suggesting the potential for an autocatalytic loop. l-Histidine, however, is a relatively complex amino acid, and requires a significant number of steps to be synthesized from the simple precursors that may have comprised primitive metabolic systems. Here, we show that a host of basic amino acids, such as diaminobutyric acid, can promote HER and CRR to a significantly greater degree than histidine despite having simpler structures. Our results demonstrate the potential of simple amino acids to augment heterogeneous catalysis, and suggest that positive feedbacks, where simple organics promote their own production by enhancing HER and CRR chemistry at the mineral surface, could have emerged readily.

## Materials and Methods

### Synthesis of Violarite Precursor

All reagents were purchased from commercial sources and used without further purification. Synthesis of violarite precursor was performed under ambient temperature and pressure. The precursor was prepared by slowly adding a solution of sodium di-*n*-butyldithiocarbamate (15.159 g) dissolved in 51 mL of ultrapure water (upw) to a solution of FeCl_3_·6H_2_O (0.901 g), NiCl_2_·6H_2_O (1.5845 g) dissolved in 50 mL of upw with continuous stirring of both solutions. After the two solutions were combined, they were stirred for an additional 1–2 h before extracting the violarite precursor by filtration. The sample was then placed in a vacuum chamber until dry. The procedure was adapted with modifications from a previously published method for greigite (Fe_3_S_4_) synthesis.^16^

### Synthesis of Violarite

Synthesis of violarite was performed using a reflux method under a nitrogen environment. First, 80 mL of oleylamine and 0.669 g of dry precursor were mixed together (suspension A). Then, 20 mL of oleylamine and 0.5921 g of tetraethylthiuram disulfide were mixed together (solution B). Suspension A was poured into a three-neck flask connected to a thermometer, a condenser, and a heater with an autotuning function for stable temperature control, and maintained under a constant flow of nitrogen. Suspension A was then heated to 230 °C, and the fully dissolved solution B was poured into the flask. The temperature was maintained at 230 °C for 1 h, after which the heater was turned off, and the suspension was allowed to cool to room temperature under nitrogen. The cooled suspension was then centrifuged at 6000 rpm for 5 min to separate the violarite product from the liquid phase. The violarite product was then washed and repelleted as above with hexane and then a 50/50 mixture of hexane and ethanol. Finally, the synthesized violarite was washed with ethanol, filtered, and stored under vacuum until dry.

### Characterization of Violarite

X-ray diffraction (XRD) analysis was performed to determine the crystalline structure and phase composition of the violarite samples. The samples were ground into a fine powder with a mortar and pestle and then ultrasonicated in isopropyl alcohol until visually dispersed. The violarite suspension was then loaded onto a silicon plate sample holder. XRD patterns were collected on RIGAKU Miniflex with Cu *K*_α_ radiation (λ = 1.5406 Å). The diffraction data were collected over a 2*θ* range of 10° to 90°. Inductively coupled plasma optical emission spectroscopy (ICP-OES) was used to quantify the elemental composition of the violarite samples. Chemical composition data were obtained on an Agilent 5100 ICP-OES. The sample was prepared by dissolving violarite powder in 2 mL of concentrated HCl. The solution was then diluted to 10 mL with upw. The elemental concentrations of Fe, Ni, S, and other trace elements were measured. Morphology images of the violarite samples were collected on a Hitachi High-Tech SU-9000 field emission scanning electron microscope (FE-SEM). X-ray photoelectron spectroscopy (XPS) was used to determine the surface composition of the violarite samples. Data were collected on ULVAC-PHI VersaProbe. XPS measurements were performed using a monochromatic Al *K*_α_ X-ray source operating at 15 kV and 25 W, with an analysis area defined by a 100 μm aperture. Charge compensation was applied using a neutralizer during data acquisition. Survey and spectra were collected with energy scanning enabled. For surface cleaning and depth profiling, Ar^+^ ion sputtering was conducted at an acceleration voltage of 10 kV over a 2 × 2 mm^2^ area. The sputtering–analysis cycle was repeated three times. A full characterization of the violarite synthesized in this work is presented in Figure S1. Briefly, the XRD pattern confirms the presence of well-defined violarite phases, with no significant impurities detected. XPS and ICP measurements confirm that nickel, iron, and sulfur are present in stoichiometrically accurate proportions.

### Preparation of the Electrode

The electrodes were prepared by cutting carbon paper into 1.5 cm × 3 cm rectangles. The carbon paper pieces were then washed twice: first, by sonication in a solution of 50/50 ultrapure water/ethanol for 5 min and then by sonication in ultrapure water for 5 min. After washing, the carbon paper pieces were dried in an oven at 40 °C. The catalyst ink was prepared by mixing 1 mg of violarite powder with 250 μL of Nafion dispersion binder diluted 20-fold with water and neutralized with 0.1 M NaOH. The prepared catalyst ink was then drop-casted onto the dried carbon paper. The electrodes were then heated again in an oven at 40 °C for an hour until dry.

### Electrochemistry

A typical H-type electrochemical cell was used, with each chamber having a volume of 60 mL and being separated by a Nafion membrane. The experimental setup is illustrated in Figure S2. The electrolyte for the experiment was 0.1 M KHCO_3_, with 10 mM amino acid added. The solution was sparged with CO_2_ for 30 min. 40 mL of electrolyte was added to each chamber. A three-electrode system was employed, with the working electrode being the violarite-dropped electrode, the reference electrode being Ag/AgCl (sat. KCl), and the counter electrode being a Pt plate with the same dimensions as the carbon paper. Chronocoulometry was then performed with a constant potential of −1.5 V (vs Ag/AgCl) for 30 min.

### Surface Analysis

In situ attenuated total reflection (ATR) infrared spectra were collected on a JASCO FT/IR-6000 with an Mercury cadmium telluride (MCT) detector. The spectra were taken in the wavenumber range of 500–4000 cm^–1^. Spectra were collected under experimental conditions similar to the electrochemical experiments, but with D_2_O instead of H_2_O as the primary solvent. Two amino acid concentrations were analyzed: 10 mM amino acid in 0.1 M KHCO_3_ and 30 mM amino acid in 0.5 M KHCO_3_. A step potential scan was conducted in the range of 0 V to −2.0 V for all in situ Fourier Transform Infrared (FT-IR) spectroscopy.

### Gas Chromatography

The gaseous products were analyzed using a gas chromatograph equipped with a dielectric-barrier discharge ionization detector (Tracera 2010, Shimadzu) using a Shinhwa Micropacked–ST column, with the exception of H_2_, which was analyzed using a gas chromatograph equipped with a thermal conductivity detector (GC-2014AT, Shimadzu). After the electrochemical experiments, the gas products were collected via a syringe and then injected into the gas chromatograph for concentration measurements.

## Results and Discussion

### Primitive Basic Amino Acids

We have previously demonstrated that the proteinogenic amino acid l-His can modestly enhance violarite-catalyzed CRR.^17^ Although the role of l-His is not fully understood, we hypothesize that l-His modulates the selectivity and activity of CRR by forming a coordination structure at the mineral-electrolyte interface. Specifically, l-His may coordinate to metallic surface sites via the amino and carboxylate groups, whereas the imidazole group interacts with CO_2_. Such an arrangement could facilitate CO_2_ adsorption and stabilize key reaction intermediates. We hypothesized that other basic amino acids could perform similar roles.

The prebiotic availability of l *-*His, however, is somewhat controversial due to the relative complexity of its imidazole side chain, and poor or absent production in many prebiotic chemistry experiments.^18^ Among various basic amino acids found in biology, including l-Arg, l-Lys, l-Orn, l-DAB, and l-His, l-His is predicted to be the most difficult to synthesize if primitive metabolic networks were similar in structure to contemporary ones. The reductive TCA (rTCA) cycle, considered to be one of the most ancient CO_2_ fixation pathways,^19^ forms an autocatalytic cycle whose intermediate metabolites are used as precursors for amino acid synthesis (Figure 1A). l-Orn and l-DAB can be synthesized from rTCA intermediates (α-keto glutarate and oxaloacetate, respectively), and may have been part of the primitive genetic code.^20^ l-DAB can also be synthesized from Gly, which is supplied from an intermediate in the Wood–Ljungdahl (WL, or reductive acetyl-CoA) pathway—another ancient CO_2_ fixation pathway. On the other hand, l-His is not synthesized directly from an rTCA intermediate, as the other basic amino acids are, and all known synthesis pathways require at least 10 steps from phosphoribosyl pyrophosphate.^21^

**Figure 1:**
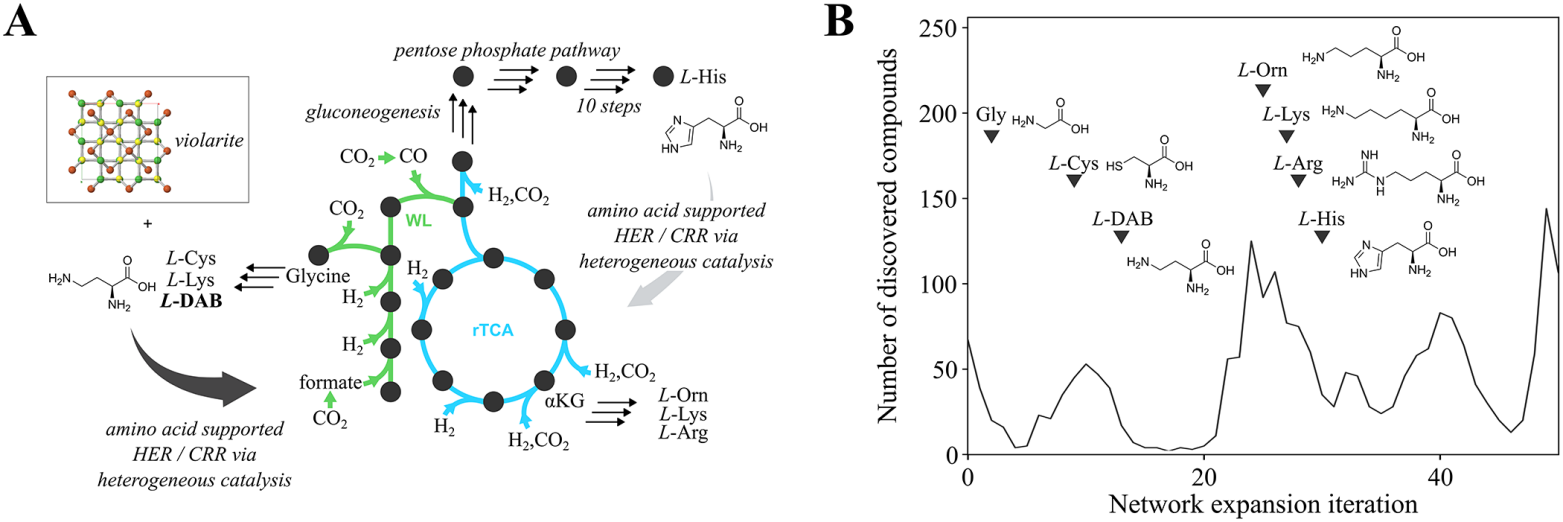
Basic amino acid-supported HER/CRR via violarite heterogeneous catalysis may have conferred robustness to prebiotic carbon fixation pathways. (A) Proposed model of primitive carbon fixation metabolism based on the reductive TCA (rTCA, blue) and Wood–Ljungdahl (WL, green) pathways. In extant life, these pathways rely on redox cofactors (ferredoxin, NAD­(P)­H, and FADH) for their reducing potential and pterin cofactors (THF and H4MPT) for carbon transfer which, in primitive life near hydrothermal vents, may have been supplied by H_2_ gas from the environment^19,31,32^ and inorganically catalyzed prebiotic analogues,^33^ respectively. In such an environment, basic amino acids produced from rTCA + WL intermediates may have created a positive feedback by promoting HER and CRR reactions via violarite heterogeneous catalysis, further promoting H_2_ and CO production. For this role, l-DAB has advantages over l-His in terms of catalytic potential (see Figure 2) and synthetic proximity to the rTCA + WL core. (B) Network expansion trajectory based on extant metabolic reactions from KEGG, starting from a seed set containing simple organic metabolites that assumes the existence of primitive rTCA + WL pathways. Of the basic amino acids considered in this study, the predicted emergence order of l-DAB precedes that of l-His, owing to the long synthetic pathway requiring 10 successive reaction steps from PRPP (phosphoribosyl pyrophosphate) required to make l-His. The full expansion terminates at iteration 99 (with 4316 compounds), of which the first 50 iterations are shown. Network expansion trajectory taken from Goldford et al.^9^

A compelling model of primitive metabolism proposes an rTCA-based metabolism braced by a WL-like feeder pathway (Figure 1A), as such a setup would benefit from both the autocatalytic benefits of rTCA and the stability benefits of a linear feeder pathway.^19^ Amino acid synthesis within this context, then, may provide a useful point of comparison with which to judge the relative accessibility and potential for high yields. To this end, we applied a recently developed model of metabolic evolution based on known biological metabolism^9^ (Figure 1B). This model hypothesizes that the network of reactions and cofactor requirements of primitive biology was similar to that of contemporary biology. Consistent with earlier analyses, this model positions l-DAB and l-Orn as potentially being the most ancient among the biological basic amino acids, a perspective that is supported by analysis of prebiotic chemistry.^22^ On the basis of this analysis, we proceeded to identify whether other basic amino acids with simpler structures that are more central to the core of carbon metabolism can significantly augment the properties of a violarite heterogeneous catalyst, as these molecules could have established early positive feedbacks between amino acid synthesis and HER/CRR.

### DAB Significantly Enhances Violarite-Catalyzed HER

As points of comparison to the basic amino acids, Gly (the simplest canonical amino acid, whose side chain consists of just a hydrogen atom) and l-Cys (a common ligand for metal and metal clusters in proteins) were also tested for their ability to augment violarite-catalyzed HER and CRR (Figure 2). We find that several amino acids do not exert significant effects on HER or CRR, including Gly (36% and −9% enhancement of HER and CRR, respectively), l-Lys (27% and −4%), and l-Arg (91% and 7%). While l-Cys had no effect on the HER (25%), it decreased the CRR activity by 67%.

**Figure 2:**
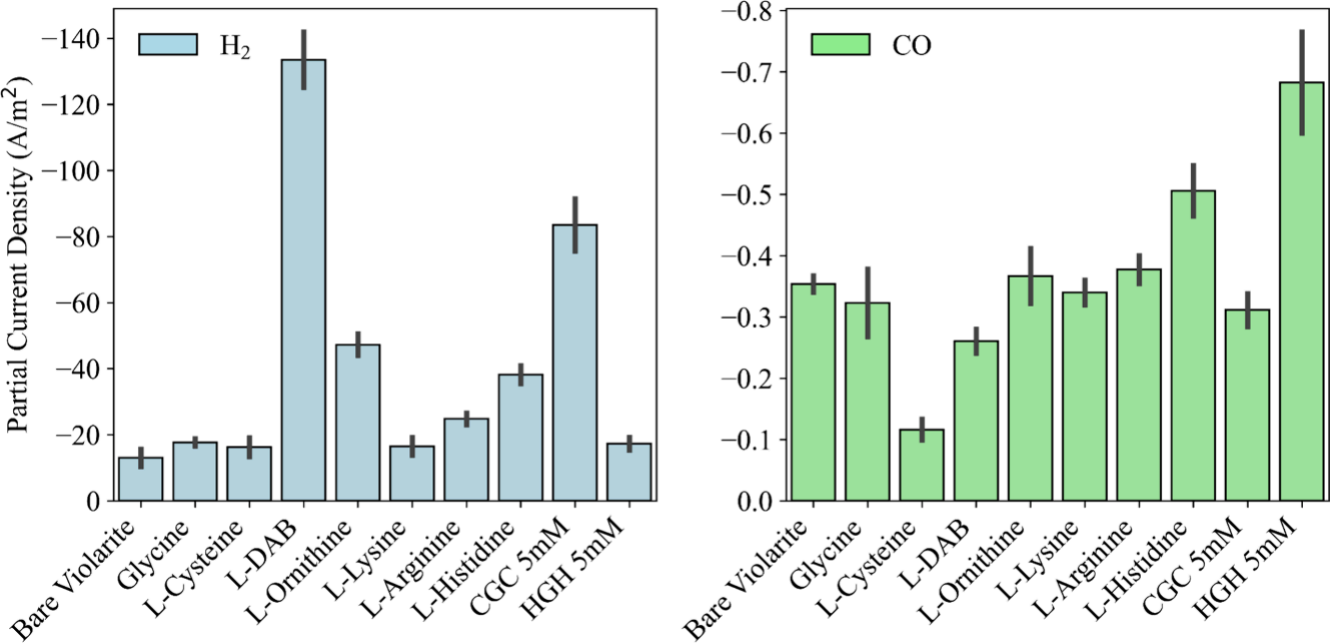
Effect of amino acids on electrochemical reduction by violarite. Production of H_2_ (left) and CO (right). The electrolyte was 0.1 M KHCO_3_ and reactions included 10 mM of the specified amino acid. CGC and HGH refer to (l-Cys)-Gly-(l-Cys) and (l-His)-Gly-(l-His) peptides, respectively, and were tested at a concentration of 5 mM peptide. Chronocoulometry was performed at a constant potential of −1.5 V (vs Ag/AgCl) for 30 min. Experimental setup as in Figure S2. Error bars indicate the standard error from three independent experiments.

Three amino acids had notable positive effects on either HER and/or CRR catalysis: l-His (194% and 43% for HER and CRR, respectively; as observed previously), l-Orn (264% and 4%), and l-DAB (928% and −26%). The effects on HER and CRR tend not to be positively correlated, and only l *-*His yielded a modest increase in both activities. Among the amino acids tested, by far the most dramatic effect was observed upon the addition of l-DAB to HER. Comparison of DAB with l-Orn and l-Lys, which have side chains with 2, 3, and 4 methylene groups, respectively, suggests that the separation between the α-amino group and the side-chain amino group has a significant impact on the activity. The observed trade-offs between HER and CRR, as well as the reaction quenching behavior of some amino acids, suggest that mixtures of small organics may have distinct properties. Thus, while our results emphasize the potential feedback induced by a single, simple amino acid, we note that primitive systems would likely have been promoted or constrained by the catalytic consequences of mixtures.

### Simple Peptides Are More Than the Sum of Their Parts

To determine whether simple peptides could significantly improve the observed catalytic rates, or if they behaved in approximately the same way as their component amino acids, we tested the effect of two tripeptides on violarite-catalyzed HER and CRR (Figure 2). The two tripeptides, (l-His)-Gly-(l-His) (HGH) and (l-Cys)-Gly-(l-Cys) (CGC), were chosen due to their high occurrence in metal-binding and metal-cluster-binding sites, including in ferredoxin, [Ni–Fe] hydrogenases, and carbon monoxide dehydrogenases. As the amino acids were analyzed at a concentration of 10 mM, peptides were analyzed at a concentration of 5 mM so that the concentration of the amino acid side-chain moieties (i.e., the imidazole and thiol moieties) was kept constant.

The properties of the peptides were rather unlike the properties of their composite amino acids: l-His increases HER, but HGH has virtually no effect; conversely, l-Cys has no effect on HER, but CGC results in a 500% increase in partial current density. Likewise, for CRR, l-Cys blocks activity (67% reduction), whereas CGC is largely neutral. HGH, on the other hand, increases the CRR-promoting activity by 93% relative to that of l-His. These results indicate that even short peptides can have properties that are distinct from those of their parent amino acids. Given that HGH and CGC motifs are found in metal-binding and metal-cluster-binding sites, these results may suggest a potentially rich functional space for even simple peptides. Nevertheless, the greatest effects on HER activity were observed by l-DAB, and all CRR activity changes were fairly modest relative to bare violarite (67% decrease, 93% increase).

### DAB Chirality Modulates Electron Flow between HER and CRR

Although H_2_ and CO are achiral, the α-carbon of DAB is a chiral center, and high-index surfaces of the crystal lattice, as well as surface defects, can be chiral. Thus, we tested whether the chirality of DAB impacts the observed CRR and HER activities (Figure 3). To our surprise, we observed significant differences between l-DAB and d-DAB. Whereas l-DAB strongly promotes HER (928% increase), d-DAB modestly promotes both HER and CRR (376% and 160% increase, respectively). d-DAB is even more efficient at promoting CRR than l-His or the HGH peptide.

**Figure 3:**
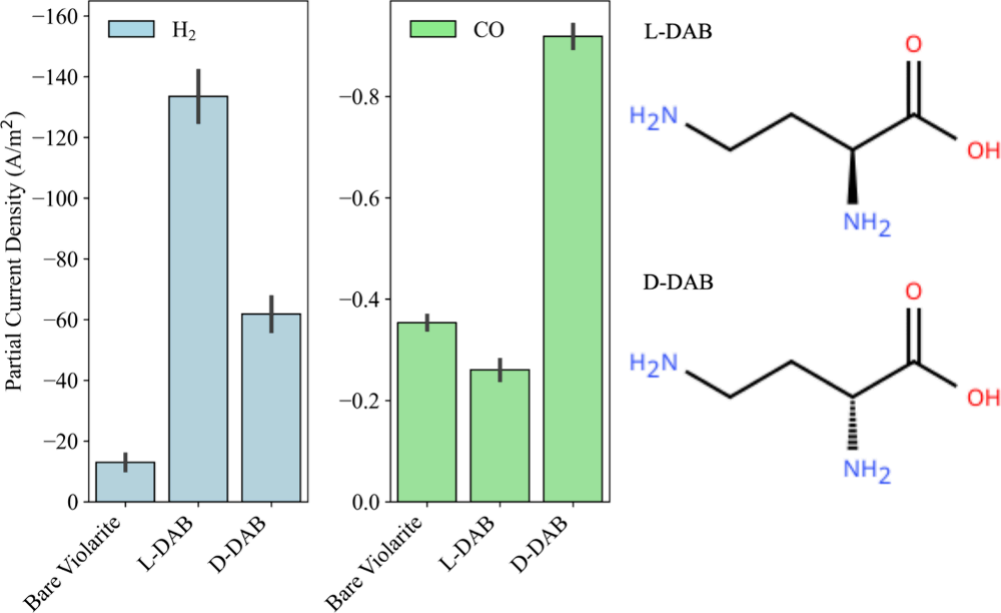
Effect of DAB chirality on electrochemical reduction by violarite. Production of H_2_ (left) and CO (right). Experimental setup as in Figure S2. Error bars indicate the standard error from three independent experiments.

The enantiomeric effects of DAB are difficult to rationalize at present. At no point during the synthesis of violarite are chiral compounds used, and the adhesive used to attach violarite to the carbon scaffold also lacks chiral centers. While a low-concentration contaminant within either the l-DAB or the d-DAB stocks (both purchased from the same supplier, Combi-Blocks, Inc.) may be the cause, it would necessitate that such a compound is exceptionally efficient at activating the violarite surface. We hypothesize that there is a subtle chiral bias in the distribution of surface defects of the violarite surface, likely imposed by an as-yet-unidentified chiral interaction during synthesis.

### DAB Has the Optimal Amino Group Spacing for HER Acceleration

First, we determined whether both amino groups of l-DAB are required for HER acceleration by testing γ-aminobutyric acid (GABA), which is missing the C_α_ amino group, and l-α-aminobutyric acid (l-AABA, also known as l-homoalanine), which is missing the side-chain amino group (Figure 4). Neither GABA nor l-AABA increases the rate of HER as much as l-DAB (173%, 64%, and 928%, respectively). Moreover, the fact that the rate enhancements produced by GABA and l-AABA are much less than 50% of that produced by l-DAB indicates that the two amino groups of l-DAB contribute to HER rate enhancement in a synergistic way. For CRR, on the other hand, both GABA and l-AABA result in an approximately 140% increase in activity relative to bare violarite, which is slightly more than the enhancement produced by l-DAB (−26%), indicating that the two amino groups have a quenching effect in this case.

**Figure 4:**
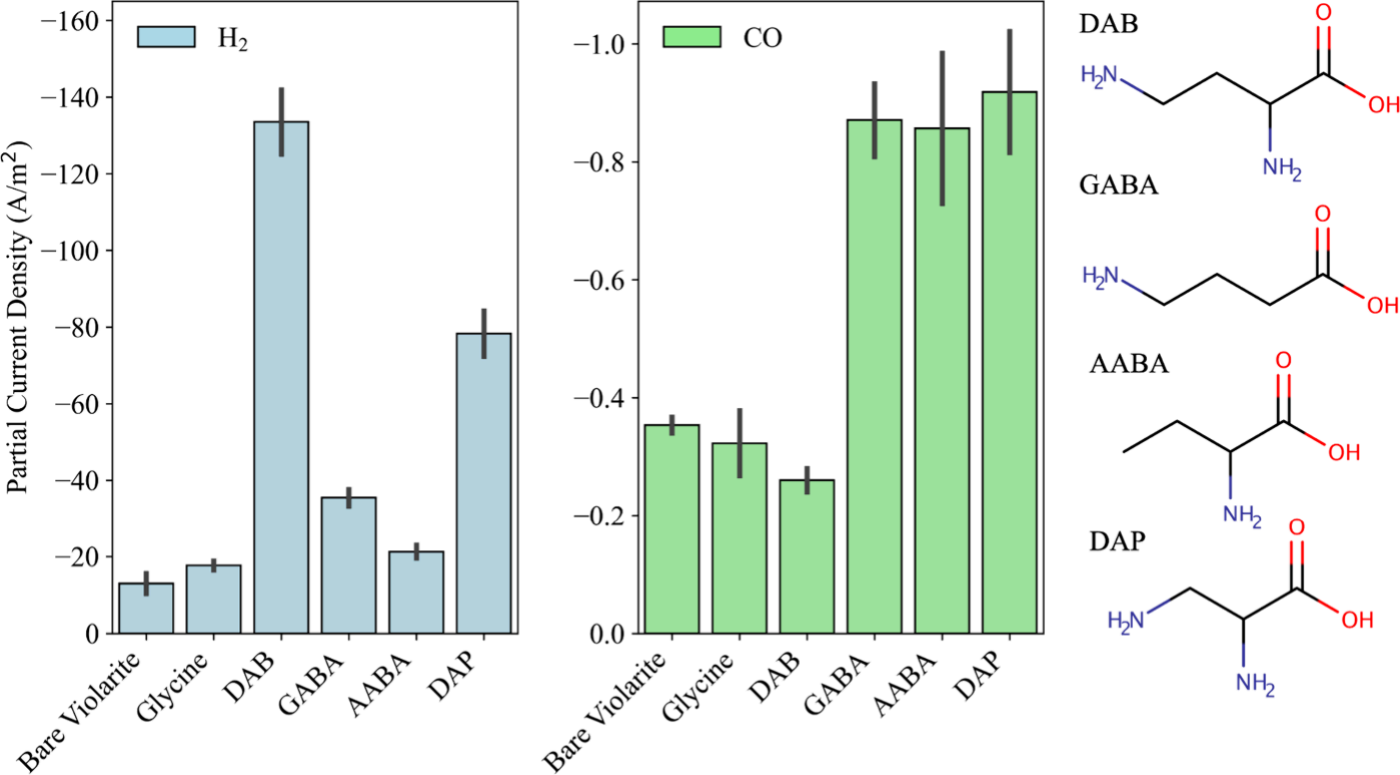
Contribution of DAB constituents to H_2_ and CO production on electrochemical reduction by violarite. Chemical structures are provided on the right. Experimental setup as in Figure S2. Error bars indicate the standard error from three independent experiments.

Previously, we observed that the spacing between the two amino groups has a significant effect on HER catalysis, with l-DAB > l-Orn > l-Lys. Does further decreasing the distance between the side-chain amino group and the C_α_ amino group result in even greater HER enhancements? We find that the HER activity enhancement from l-diaminopropionic acid (l-DAP) is less than that of l-DAB, suggesting that l-DAB has the optimal spacing for HER catalysis. CRR, on the other hand, has enhanced activity (160% improvement) in the presence of l-DAP.

These data suggest that l-DAB is a local maximum for HER rate enhancement by a simple amino acid and that HER activity is being promoted by a roughly conserved mechanism across these related amino acids, which involves the synergistic participation of both amino groups. For CRR, on the other hand, GABA, l-AABA, and l-DAP (but not l-Orn or l-Lys) increase the partial current density to about −0.8 A/m^2^. In other words, bringing the two amino groups in close proximity, or removing one or the other amino group, results in the same degree of rate enhancement over bare violarite. Given the comparatively low partial current density of CRR relative to HER, and the more modest influence of amino acids in solution, we hypothesize that HER may benefit from specific interactions with violarite, whereas CRR may be enhanced by general effects.

### Amino Acids May Regulate Proton Supply to the Violarite Surface

To clarify how small molecules augmented CRR and HER selectivity, FT-IR spectroscopy measurements were conducted (Figure 5). l-DAB, l-DAP, l-AABA, and GABA were dissolved in ultrapure water, and spectra were then collected before and after 30 min CO_2_ sparging (Figure 5A–D). Note that this experiment is in the absence of the violarite surface. All of the small molecules tested, except for l-AABA, showed the evolution of symmetric and asymmetric N–H stretching bands after CO_2_ sparging. Whereas the diamino acids l-DAB and l-DAP exhibited clear, strong peaks, GABA exhibited only weak N–H stretching bands. However, no correlation between the formation of N–H stretching peaks and CRR activity was observed: l-DAB slightly quenches CRR (−26%), whereas GABA, l-AABA, and l-DAP promote CRR to approximately the same degree (∼160%); yet, the evolution of N–H stretching peaks after sparging ranges from nearly undetectable (l-AABA) or weak (GABA) to strong l-DAP. Nonetheless, the fact that l-DAP and l-DAB both have significantly stronger N–H stretching peaks than other compounds indicates that having two amino groups strongly promotes CO_2_ binding.

**Figure 5:**
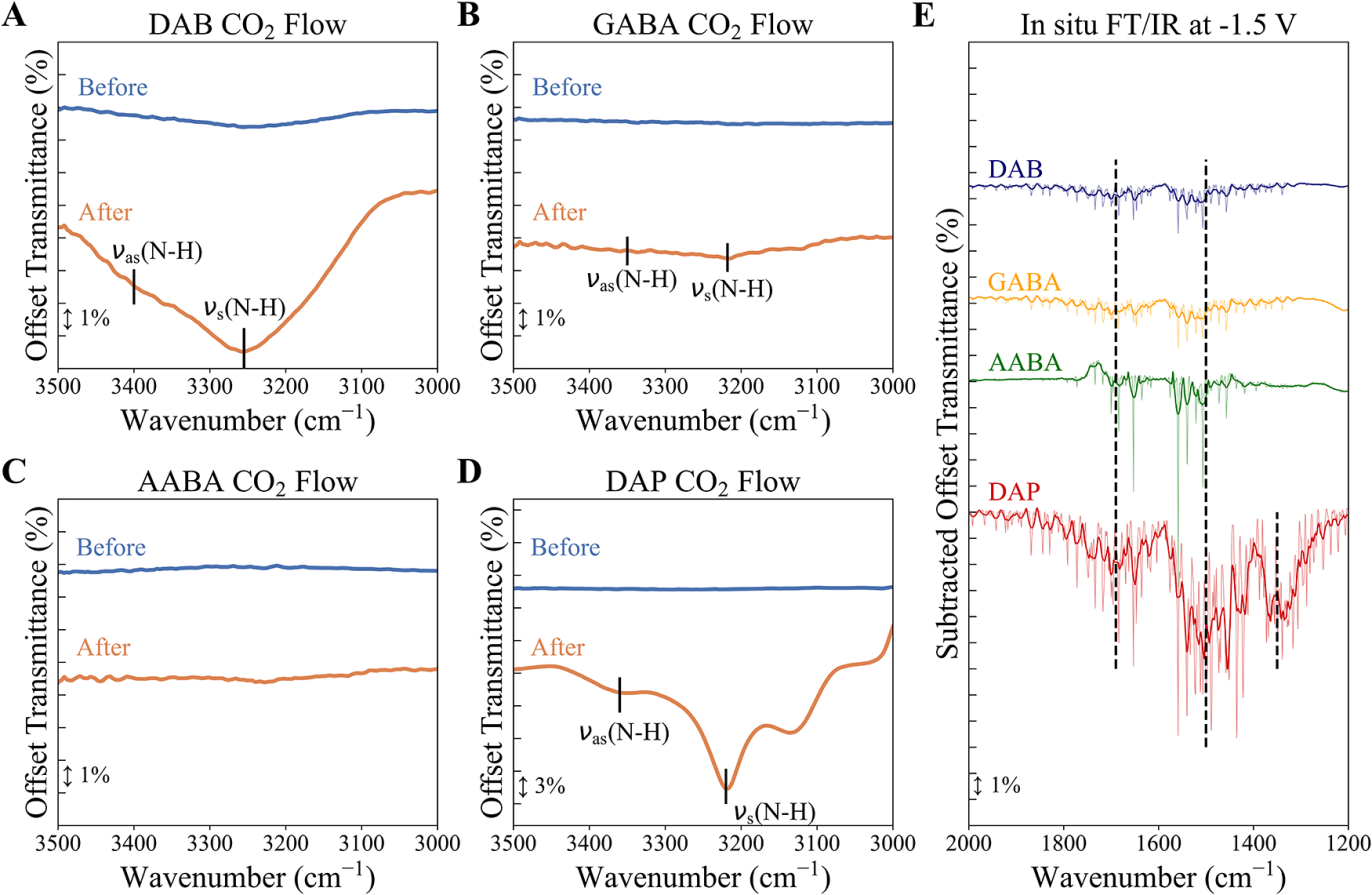
Spectroscopic analysis of interactions between CO_2_ and amino acids after CO_2_ purging. FT-IR spectra for (A) DAB, (B) GABA, (C) AABA, and (D) DAP in the absence of violarite. Indicated peaks refer to the symmetric (ν_s_(N–H)) and asymmetric (ν_as_(N–H)) stretching modes of the N–H bond. Note that DAP and DAB have the strongest indications of CO_2_ binding. (E) In situ FT-IR spectroscopy at −1.5 V in the presence of violarite. CO_2_ was purged for 30 min. All amino acids have the concentration of 10 mM.

To better understand the catalytic role of these compounds, in situ FT-IR spectra were collected in the presence of violarite (Figure 5E). Two conditions were tested: 10 mM of the small molecule in 0.1 M KHCO_3_ and 30 mM of the small molecule in 0.5 M KHCO_3_. At the lower small molecule concentration, step scans show two peaks associated with CO: A peak at ∼1700 cm^–1^ for bridge-bonded CO and a peak at ∼1530 cm^–1^ for adsorbed COO^–^ species in the presence of l-DAP. However, these peaks were not as prominent with l-DAB, GABA, and l-AABA, again suggesting a role for the side-chain amino group in CO binding. These spectra did not show any detectable coordination peaks. FT-IR spectra collected at higher concentrations (30 mM) of small molecules are shown in Figure S5. The C–O stretching band of the carboxyl group^23^ (∼1370 cm^–1^) was detected in all the spectra except for bare violarite, indicating that C–O stretching is indeed caused by the added small molecules. Another peak at ∼1640 cm^–1^, attributed to the amide I band,^24–26^ was not present in GABA, consistent with its molecular structure, or the bare mineral. We do not see evidence for oxidized or reduced amino acid products in the FT-IR measurements, suggesting that the amino acids remain intact during the electrochemical reaction.

On the basis of our observations, we propose a hypothetical model for small molecule-supported HER and CRR electrocatalysis on violarite. Three distinct factors affect the activity of HER and CRR: the existence of a side-chain amino group, the existence of an α-amino group, and the separation between them. The α-amino group and the α-carboxyl group can interact via intramolecular hydrogen bonding in the zwitterionic form of amino acids. The advantage of such a hydrogen bond network is that it can produce a proton relay, in which protons are channeled from the bulk solution to the surface of the catalyst, thereby replenishing protons for the surface reactions. We note that a proton relay could reasonably support both HER and CRR reactivities, and such explanations have been used for similar systems.^27,28^ In addition to promoting proton relay to the catalyst, the carboxyl group can bind directly to the surface of violarite, and previously we have observed that imidazole is a much weaker catalyst than l-His, in part due to a loss of surface binding. The side-chain amino group, on the other hand, may help capture CO_2_ at the surface for CRR. Once a bias is applied, and electrons flow into the system, and CO_2_ adsorption onto the violarite surface, likely as the surface-bound *COO^–^ species, is promoted. At the same time, the hydrogen bond network produced by the small molecule relays protons directly toward the mineral surface. As electrons and protons are continuously flowing toward the catalyst, *COO^–^ reacts to form *COOH, which then dissociates into *CO and *OH. Hydrogenation of *OH forms H_2_O and CO dissociates from the mineral surface.^29,30^ Computational approaches such as Density Functional Theory or Molecular Dynamics may shed light on the feasibility of the “proton relay mechanism” proposed here.

## Concluding Remarks

We demonstrate that simple basic amino acids can promote violarite-mediated electrocatalysis of HER and CRR, potentially via a proton relay-based mechanism. This result highlights how the production of simple organics via heterogeneous catalysis could have resulted in a positive feedback, in which the downstream products of HER and CRR further increase the rate of HER and CRR at the mineral surface. Finally, this result suggests that amino acids, long before their incorporation into peptides and proteins, could have played key supportive roles in early catalysis.

## Supplementary Material



## Notes

Characterization of violarite: (a) XRD pattern, (b) XPS spectrum, (c) SEM images and atomic ratio from ICP-OES measurements (Figure S1); picture of electrochemical cell used for activity measurement (Figure S2); picture of prepared electrode (Figure S3); XPS spectra of violarite powder (Figure S4); in situ FT-IR spectroscopy in the presence of 30 mM DAB, GABA, AABA, and DAP (Figure S5); ICP Ni calibration (Figure S6); ICP S calibration (Figure S7); ICP Fe calibration (Figure S8); Faradaic efficiency for all products in the presence of amino acids (Figure S9); partial current density for all products in the presence of amino acids (Figure S10); current density data of bare violarite, in the presence of Histidine, and in the presence of DAB (Figure S11); product partial current density of bare carbon paper with CO_2_, bare violarite without CO_2_, and bare violarite with CO_2_ (Figure S12) (PDF)
