# Supplementary material for: Primitive Basic Amino Acids Promote Mineral-Catalyzed Electrochemical Reduction of H+ and CO2
Source: J Phys Chem C Nanomater Interfaces. 2026 Jun 18;130(26):9040–7. doi: 10.1021/acs.jpcc.6c02384 (PMC13339756; doi:10.1021/acs.jpcc.6c02384)
Supplement: Supplementary file 1 [file jp6c02384_si_001.pdf]

**Supporting Information**

# Primitive Basic Amino Acids Promote Mineral-catalyzed Electrochemical Reduction of $H^+$ and $CO_2$

Siang Chen[1], Tatsuya Corlett[2], Norio Kitadai[3], Ryuhei Nakamura[2,4,5], Masahiro Miyauchi[1,6], Liam M. Longo[2,7*] and Akira Yamaguchi[1,4*]

[1]Department of Materials Science and Engineering, School of Materials and Chemical Technology, Institute of Science Tokyo, 2-12-1 Ookayama, Meguro-ku, Tokyo 152-8552, Japan

[2]Earth-Life Science Institute, Institute of Science Tokyo, Tokyo, 152-8550, Japan

[3]Super-cutting-edge Grand and Advanced Research (SUGAR) Program, Japan Agency for Marine-Earth Science and Technology (JAMSTEC), 2-15 Natsushima-cho, Yokosuka 237-0061, Japan

[4]Biofunctional Catalyst Research Team, RIKEN Center for Sustainable Resource Science, 2-1 Hirosawa, Wako, Saitama 351-0198, Japan

[5]Department of Chemical Science and Engineering, School of Materials and Chemical Technology, Institute of Science Tokyo, 2-12-1 Ookayama, Meguro-ku, Tokyo 152-8552, Japan

[6]Hydrogen Boride Research Center, Tsukuba Institute of Advanced Research, University of Tsukuba, 1-1-1 Tennodai, Tsukuba, Ibaraki 305-8573, Japan

[7]Blue Marble Space Institute of Science, Seattle, Washington 98154, United States.

[*]To whom correspondence should be addressed: llongo@elsi.jp, ayamaguchi@ceram.titech.ac.jp

**Table of contents**

1. Characterization of violarite: (a) XRD pattern, (b) XPS spectrum, (c) SEM images and atomic ratio from ICP-OES measurements. (Fig. S1)
2. Picture of electrochemical cell used for activity measurement. (Fig. S2)
3. Picture of prepared electrode. (Fig. S3)
4. XPS spectra of violarite powder. (Fig. S4)
5. In situ FTIR spectroscopy in the presence of 30 mM DAB, GABA, AABA, and DAP. (Fig. S5)
6. ICP Ni calibration. (Fig. S6)
7. ICP S calibration. (Fig. S7)
8. ICP Fe calibration. (Fig. S8)
9. Faradaic efficiency for all products in the presence of amino acids. (Fig. S9)
10. Partial current density for all products in the presence of amino acids. (Fig. S10)
11. Current density data of bare violarite, in the presence of Histidine, and in the presence of DAB. (Fig. S11)
12. Product partial current density of bare carbon paper w/ $CO_2$, bare violarite w/o $CO_2$ and bare violarite w/ $CO_2$. (Fig. S12)

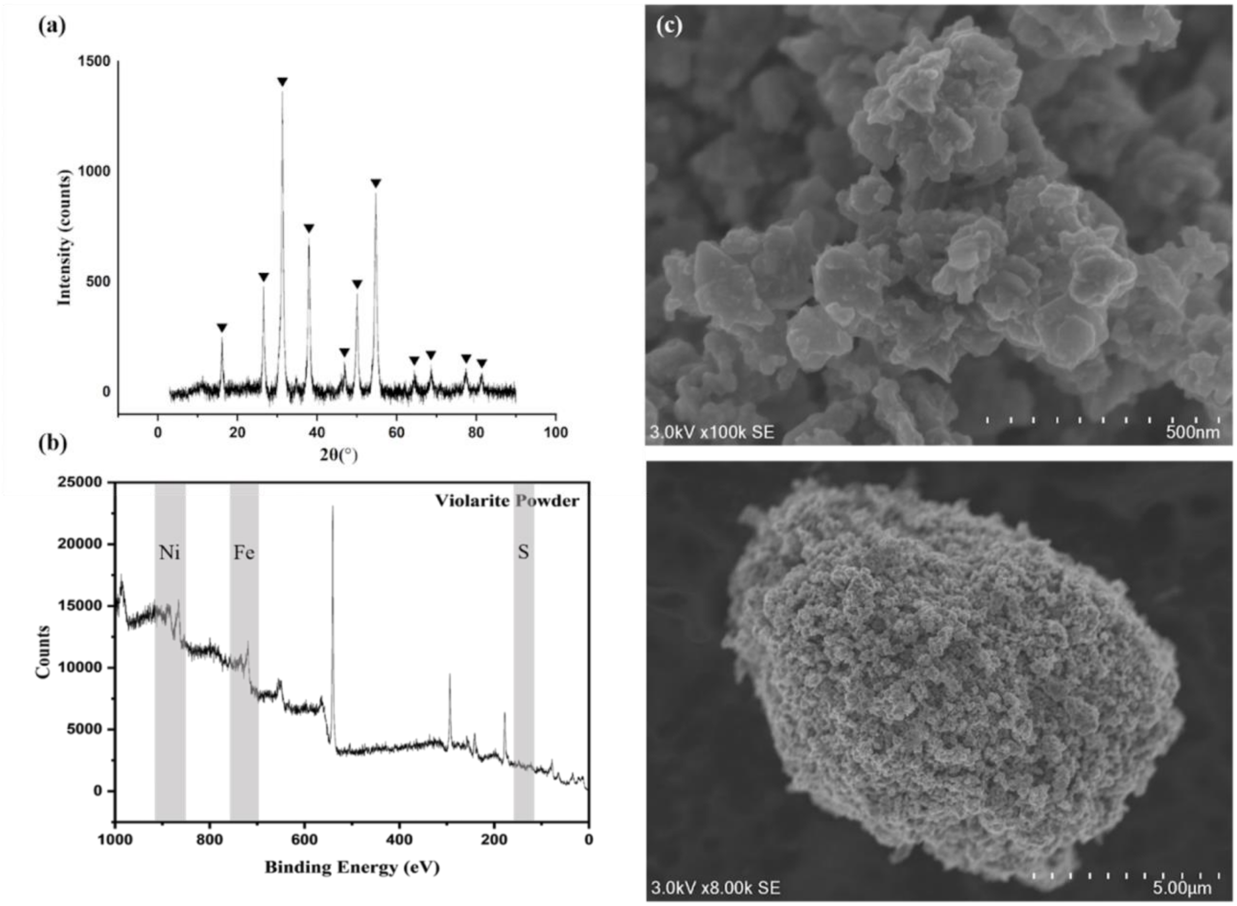


| Element | Measured Atomic Ratio | Theoretical Atomic Ratio |
|---|---|---|
| Ni | 14.3 | 14.3 |
| Fe | 28.3 | 28.6 |
| S | 57.3 | 57.1 |

**Figure S1**. Characterization of violarite: (a) XRD pattern, (b) XPS spectrum, (c) SEM images and atomic ratio from ICP-OES measurements.

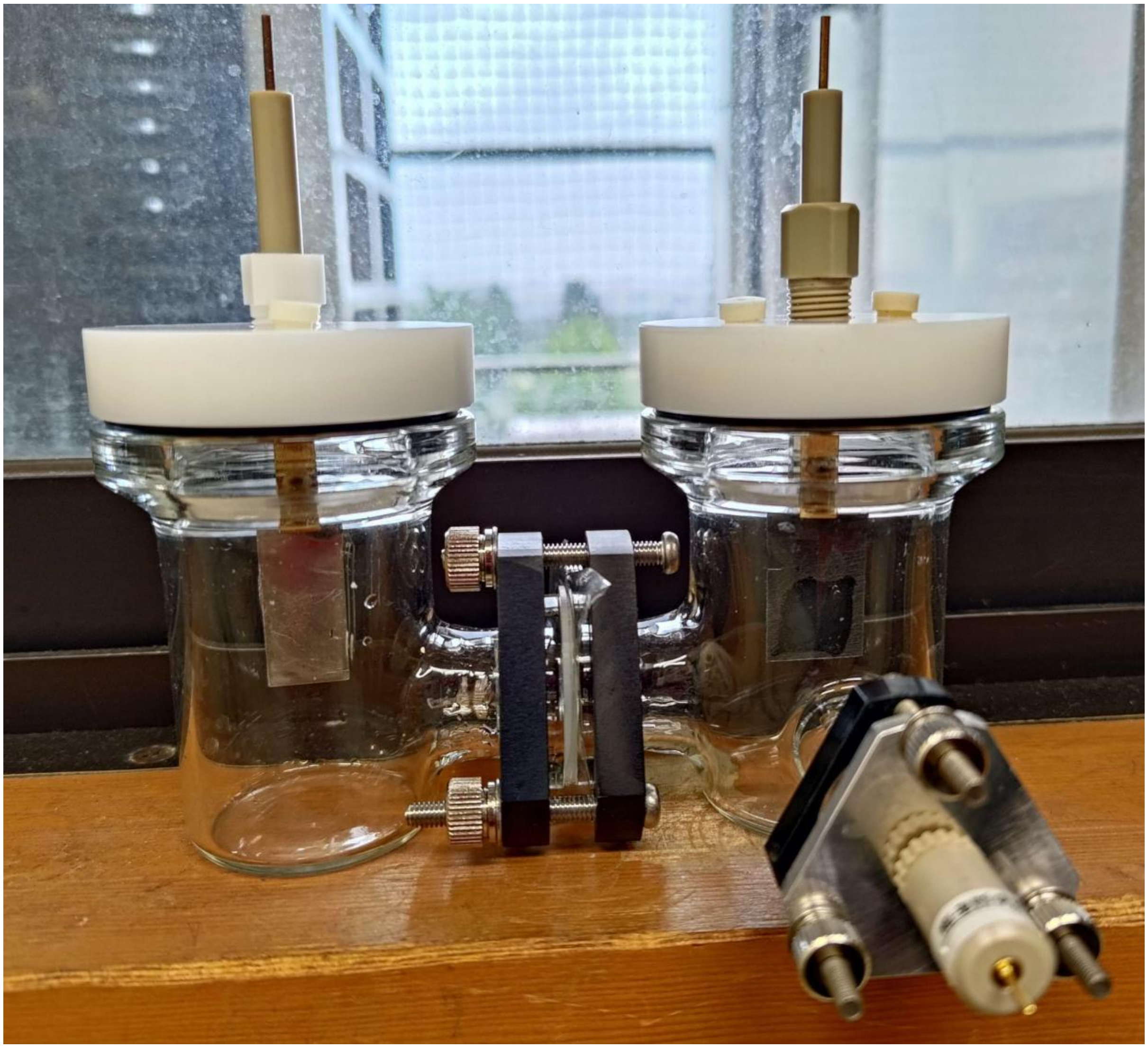

**Figure S2**. Picture of experimental setup.

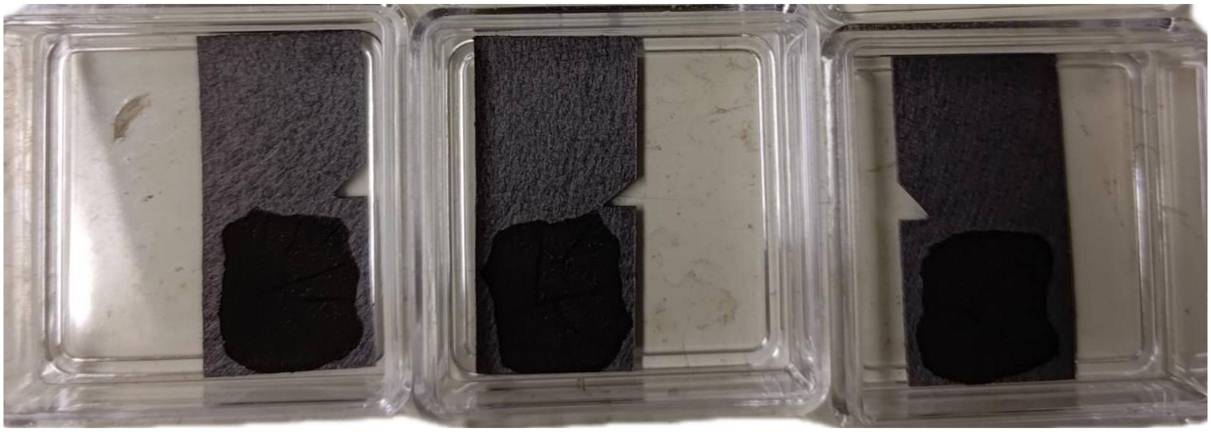

**Figure S3**. Picture of prepared electrode.

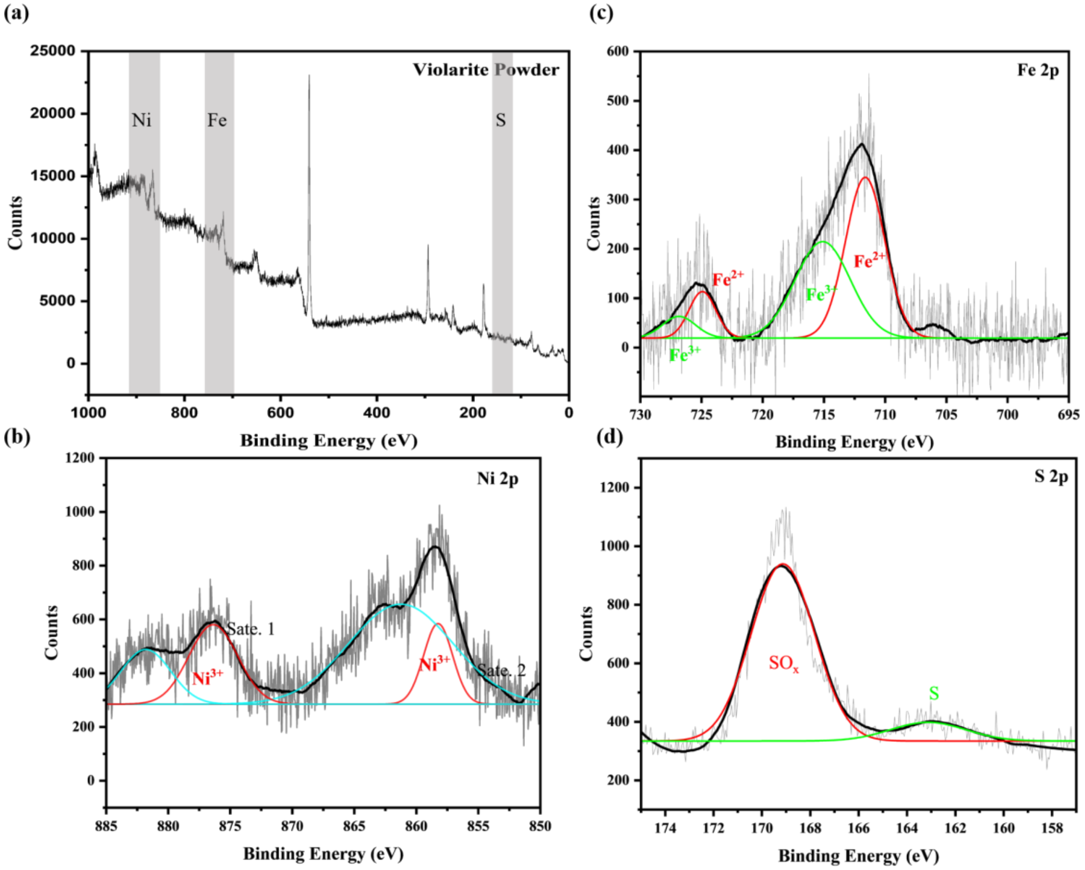


**Figure S4**. XPS spectra of violarite powder: (a) survey spectrum, (b) Ni 2p, (c) Fe 2p and (d) S 2p.

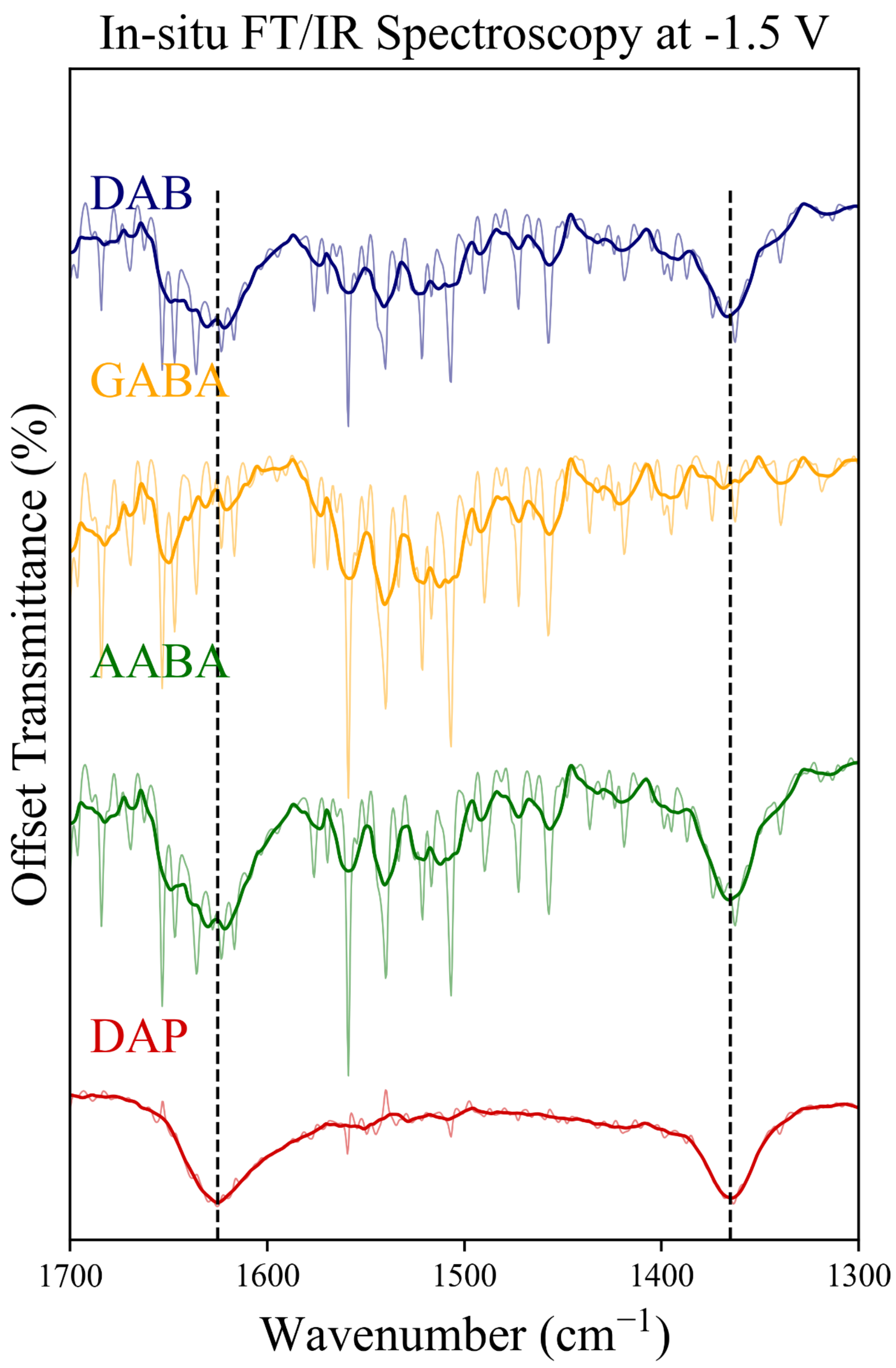


**Figure S5**. In situ FTIR spectroscopy in the presence of 30 mM DAB, GABA, AABA, and DAP.

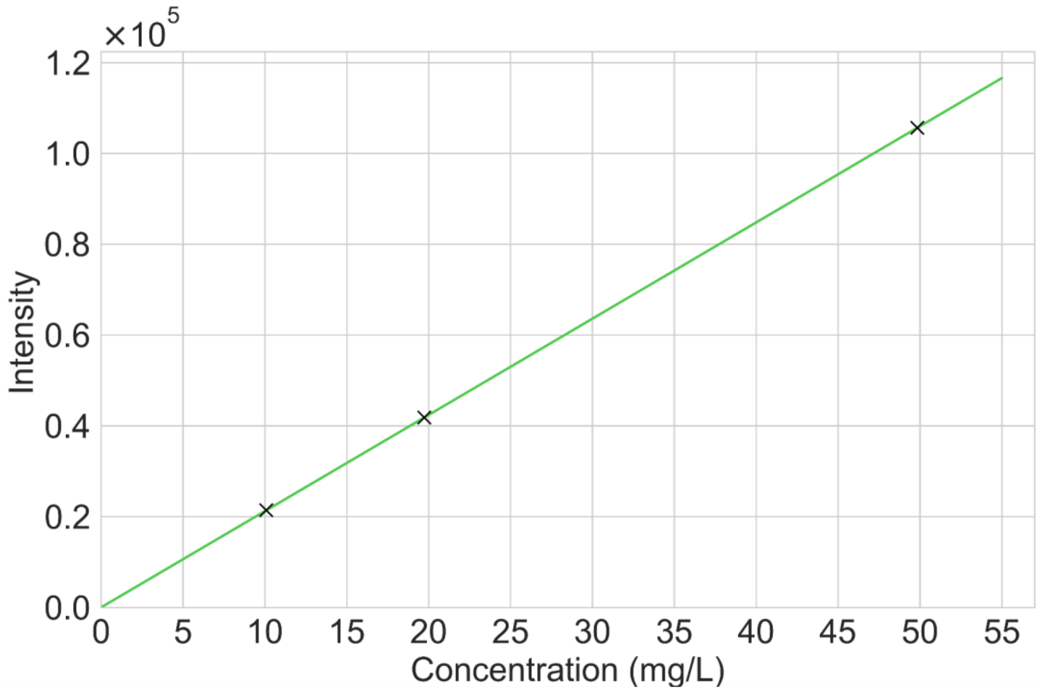


**Figure S6.** ICP Ni calibration.

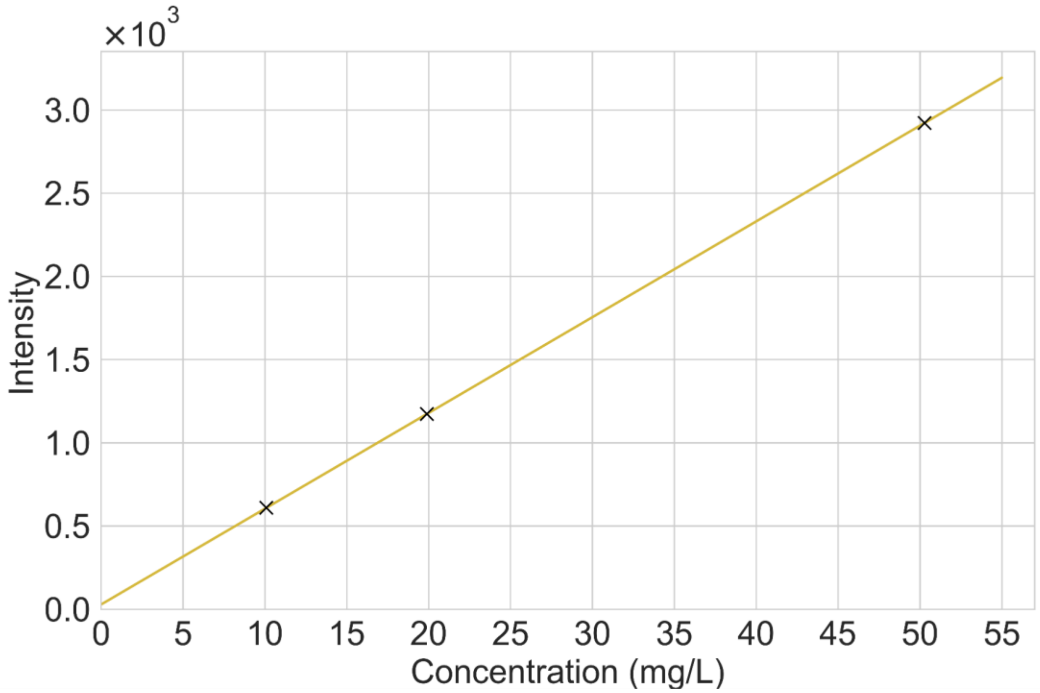


**Figure S7.** ICP S calibration.

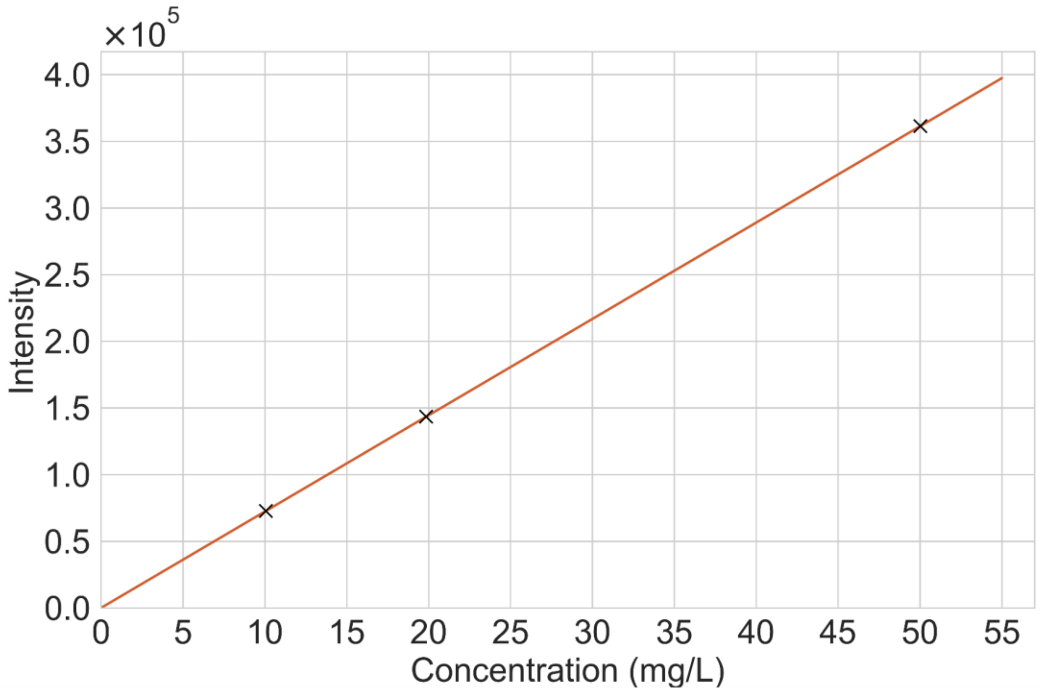


**Figure S8**. ICP Fe calibration.

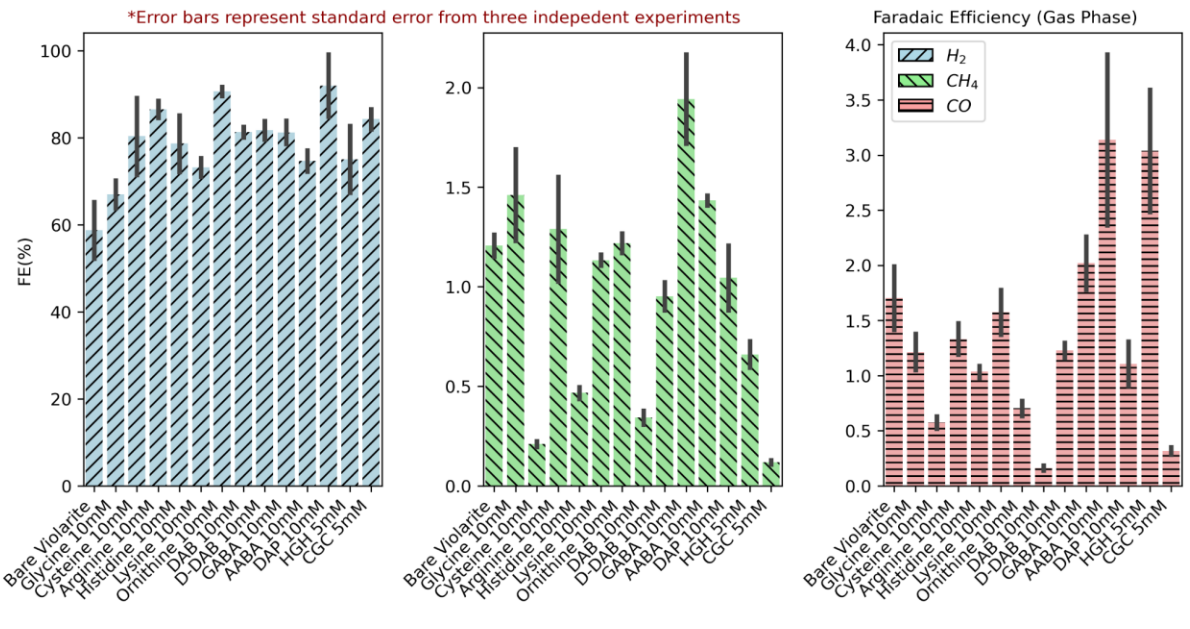


**Figure S9**. Faradaic efficiency for all products in the presence of amino acids.

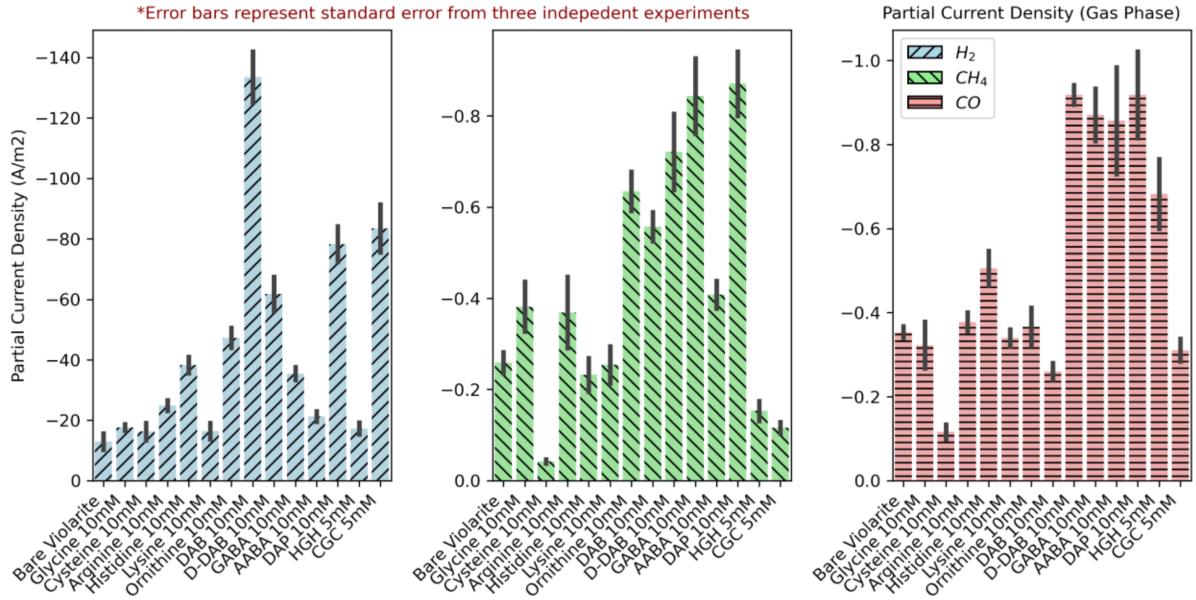


**Figure S10**. Partial current density for all products in the presence of amino acids.

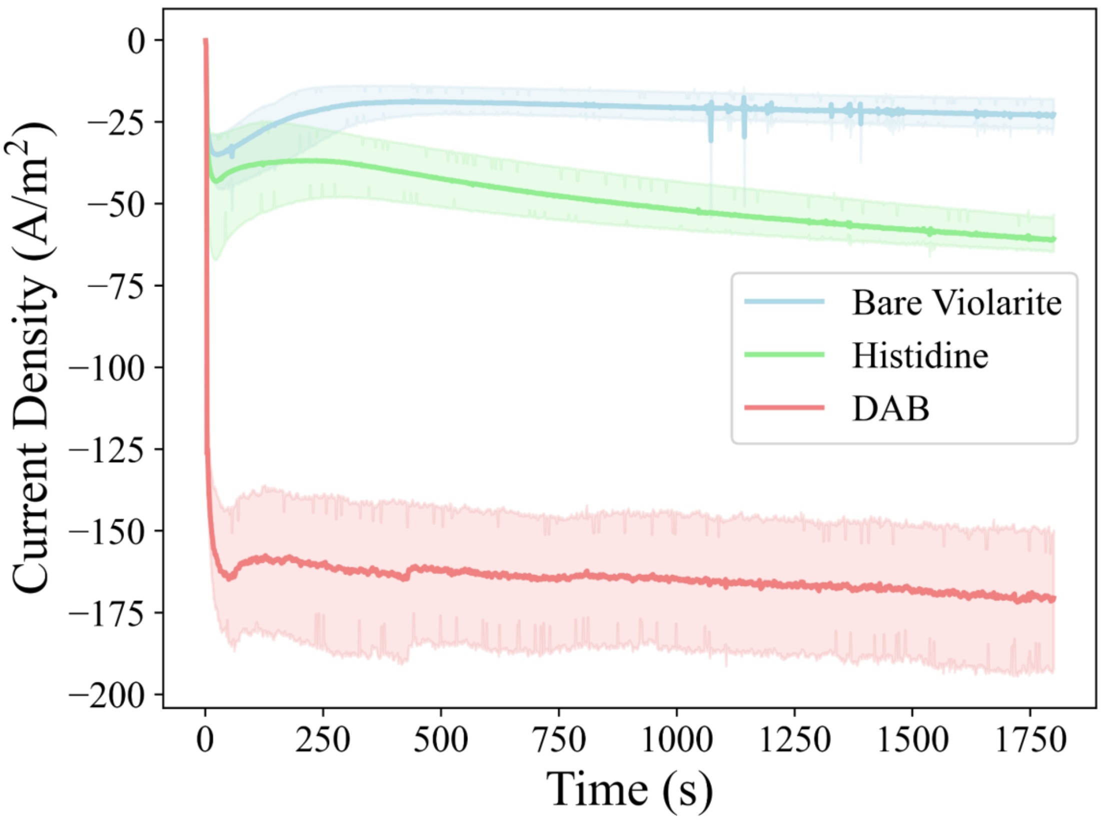


**Figure S11.** Current density data of bare violarite, in the presence of Histidine, and in the presence of DAB.

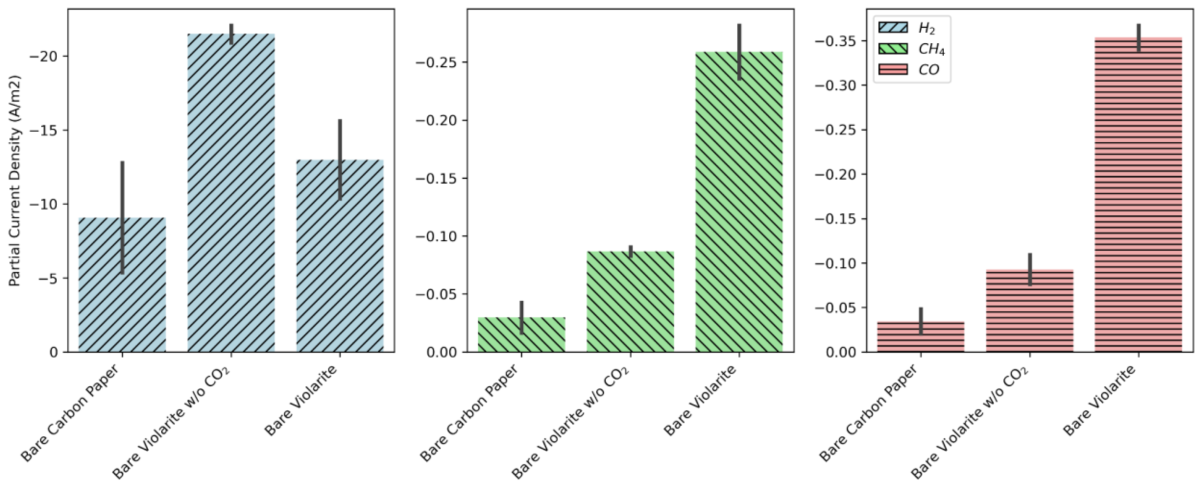


**Figure S12**. Product partial current density of bare carbon paper w/ $CO_2$, bare violarite w/o $CO_2$ and bare violarite w/ $CO_2$.
